# Diagnostic and Management Challenges in a Non-Infectious Pacemaker Pocket Swelling: A Case Report and Literature Review

**DOI:** 10.3390/diagnostics16183031

**Published:** 2026-09-18

**Authors:** Ștefan Horia Roșian, Samira Smailović, Irina Sandu, Oana Gabriela Șomcutian, Adela Nicoleta Roșian, Alexandru Oprea, Paul-Mihai Boarescu, Alexandra Dădârlat-Pop

**Affiliations:** 1 Faculty of Medicine, “Iuliu Haţieganu” University of Medicine and Pharmacy, 8 Victor Babeş Street, 400012 Cluj-Napoca, Romaniadadarlat.alexandra@yahoo.ro (A.D.-P.); 2 Cardiology Department, Heart Institute “Niculae Stăncioiu”, 19-21 Moţilor Street, 400001 Cluj-Napoca, Romania; 3 Radiology Department, Heart Institute “Niculae Stăncioiu”, 19-21 Moţilor Street, 400001 Cluj-Napoca, Romania; 4 Department of Pathology, Heart Institute “Niculae Stancioiu”, 19-21 Moţilor Street, 400001 Cluj-Napoca, Romania; 5 Cardiovascular Surgery Department, Heart Institute “Niculae Stăncioiu”, 19-21 Moţilor Street, 400001 Cluj-Napoca, Romania; 6 Cardiology Department, Clinical Emergency County Hospital “Saint John the New”, 720229 Suceava, Romania; 7 Department of Medical-Surgical and Complementary Sciences, Faculty of Medicine and Biological Sciences, “Ştefan cel Mare” University of Suceava, 720229 Suceava, Romania

**Keywords:** cardiovascular diagnosis, cardiac imaging, tissue characterisation, leadless pacemaker, CIED pocket swelling

## Abstract

**Background and Clinical Significance:** The widespread use of cardiac implantable electronic devices (CIEDs) has led to an increasing number of pocket-related complications. While early hematoma and infection are common, late-onset non-infective cystic pocket collections are rare and may closely mimic infectious processes, creating significant diagnostic and therapeutic dilemmas. **Case summary:** We report the case of an 81-year-old woman with a complex medical history (including a previous device-related infection requiring complete pacing system extraction), with a gradually enlarging, painless elastic mass at the secondary pacemaker pocket site, without local inflammatory signs, with normal inflammatory markers, and repeated negative blood cultures. Imaging demonstrated a large cystic collection surrounding the pacemaker. Surgical exploration was performed and a large volume of odourless, thick, yellowish fluid was evacuated from a fibrous capsule. Complete extraction of the pacing system was undertaken. Fluid microbiological cultures were negative and histopathological analysis revealed a chronic, nonspecific fibro-inflammatory granulomatous process. A leadless transcatheter pacemaker was implanted with favourable clinical and procedural outcomes. **Conclusions:** This case highlights the complexity of decision-making in a late-onset large fluid accumulation within the device pocket when clinical and microbiological evidence of infection is lacking, and the etiology remains uncertain. The local tissue findings, after the pocket has been opened and drained, along with the distinct clinical characteristics of each case, must ultimately define the therapeutic strategy. In high-risk patients with diagnostic uncertainty, leadless pacing systems provide an effective strategy for definitive rhythm management while minimising the risk of future infection.

## 1. Introduction

The use of cardiac implantable electronic devices (CIEDs), including permanent pacemakers and implantable cardioverter-defibrillators (ICDs), has increased substantially over the past few decades, leading to parallel growth in device-related complications [1,2,3].

Very few data on the incidence and risk of late-onset CIED complications detected after discharge have been published to date. The overall incidence of complications is better known, being estimated around 5–6%, and 3–4% for major complications, respectively [1,4]. The complication risk is higher within the first two months after CIED implantation (12.4%) [5]. Late-stage pocket issues occur months to years after implantation and principally encompass device erosion, infection, and skin changes, especially common in elderly, frail patients [2,6,7,8].

The most common causes of pocket swelling include early postoperative hematoma and pocket infection [9,10], whereas delayed-onset, non-infectious cystic collections remain distinctly uncommon [11]. Such late complications may closely mimic infectious processes, resulting in potential overtreatment, including complete system extraction, and represent a real diagnostic challenge [12,13,14].

In addition to antibiotic therapy, the mandatory treatment of definite systemic or localised CIED pocket infections is complete removal of all parts of the system, including the device and all transvenous leads (active, abandoned, epicardial, lead fragments) and meticulous debridement of the device pocket with complete excision of the fibrotic capsule as well as removal of all non-absorbable suture material [9].

Less clear is the management in cases of non-infectious etiology, involving multiple risk factors and/or a history complicated by infections and repeated reinterventions within the same anatomical area. In these situations, the operator’s experience makes a distinct difference. Accurate assessment of the local anatomy, even in the absence of infection, alongside the patient-specific clinical factors, is crucial. Furthermore, the subsequent decision regarding the type of CIED to be implanted following collection drainage is equally important. In the era of non-transvenous systems, leadless pacemakers represent an excellent alternative in such situations.

## 2. Detailed Case Description

We describe the case of an 81-year-old woman who presented with progressive exertional fatigue and a gradually enlarging swelling located at the pacemaker site, first noted in February 2024. The swelling increased steadily and gradually over several months but remained painless. Her medical history was significant for dilated cardiomyopathy with preserved ejection fraction, grade II mitral regurgitation, sinus node disease, seropositive rheumatoid arthritis, bilateral Methicillin-resistant *Staphylococcus aureus* (MRSA) septic arthritis of the knees in the past, and a left humerus fracture with malunion. Device history started with the implantation of a primary dual-chamber pacemaker in 2018 for sinus node disease, followed in 2019 by leads and device extraction due to MRSA infective endocarditis involving the right ventricular lead and the aortic valve, successfully treated with a specific targeted antibiotic regimen at that time. A new dual-chamber system was subsequently implanted in 2021, also in the infraclavicular left side.

**Figure 1:**
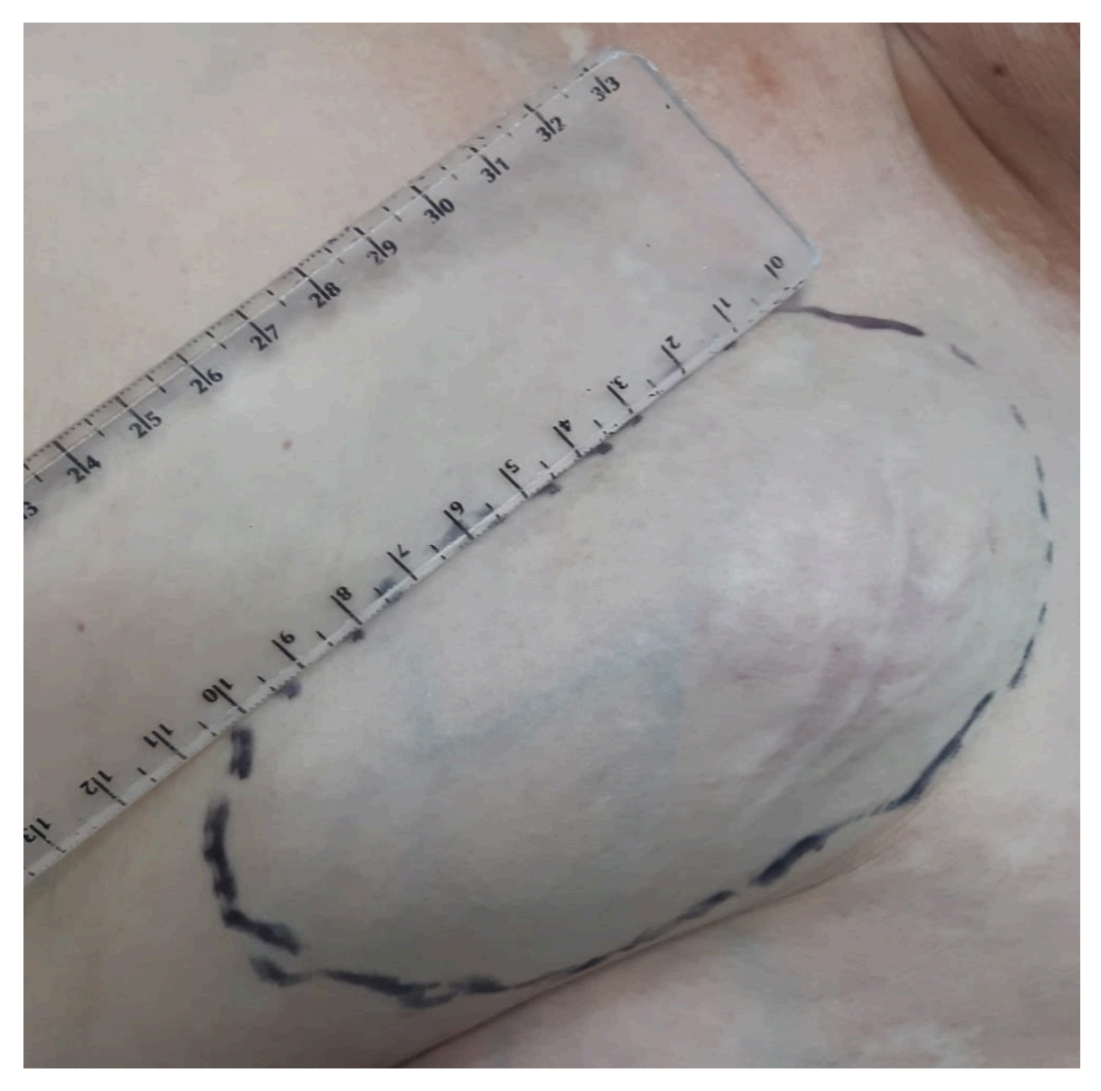
Clinical appearance of the gradually enlarging, irregular, painless, elastic mass (10 × 10 cm) located at the pacemaker site, extending toward the left axilla without cutaneous inflammatory changes.

Clinically, the patient denied fever, chills, local pain, erythema, or drainage from the pocket site. Examination revealed a large, irregular, elastic mass of approximately 10 × 10 cm over the left pre-pectoral region, extending toward the axilla (Figure 1). The swelling was only mildly tender and not associated with cutaneous inflammatory changes. Interrogation of the dual-chamber pacemaker demonstrated stable function, with normal lead parameters and approximately 80% ventricular pacing burden.

Peripheral blood investigations revealed no serum inflammatory markers, including C-reactive protein. Thyroid function tests were within normal limits. Multiple sets of blood cultures for aerobic, anaerobic, and fungal organisms were negative.

Transoesophageal echocardiography visualized the atrial lead, including its implantation site in the right atrial appendage, without any adherent masses. On the atrial portion of the ventricular lead, a highly mobile, filamentous structure measuring approximately 5 mm was identified. No additional suspicious intracardiac masses were noted. Valvular assessment did not reveal echocardiographic evidence of mitral or aortic vegetations. Ultrasound of the soft tissues demonstrated a large anechoic cystic subcutaneous collection containing clear fluid.

**Figure 2:**
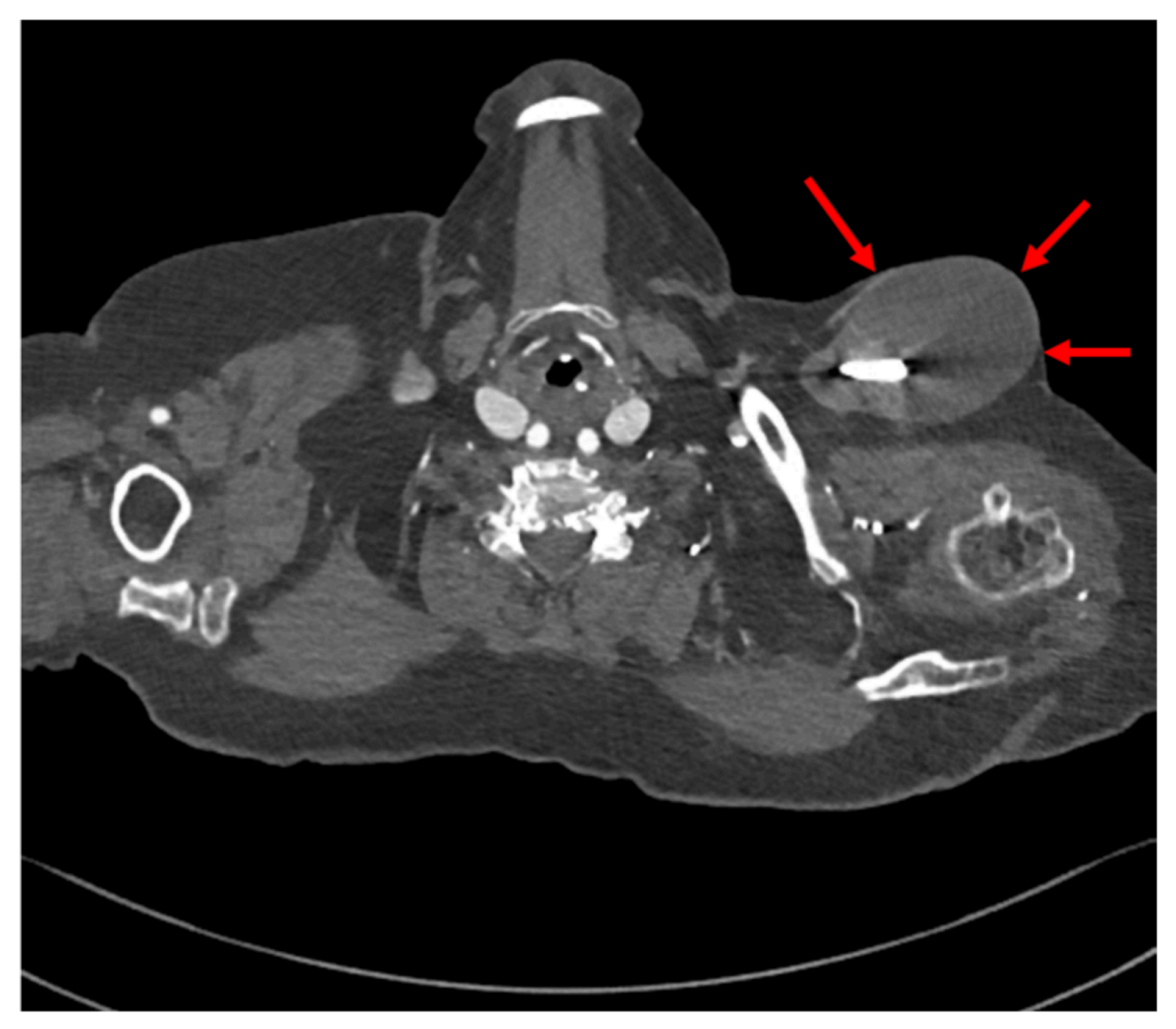
Thoracic computed tomography (CT) angiography showing a voluminous, thin-walled cystic lesion of liquid density (11 × 5 × 6.8 cm; 200 mL volume) encapsulating the pacemaker and proximal leads (red arrows).

Thoracic computed tomography (CT) angiography was performed to better characterize the structure. The imaging revealed a voluminous, thin-walled cystic formation of liquid density, measuring approximately 11 × 5 × 6.8 cm, with a 200 mL volume, encapsulating the pacemaker and proximal leads (Figure 2).

**Figure 3:**
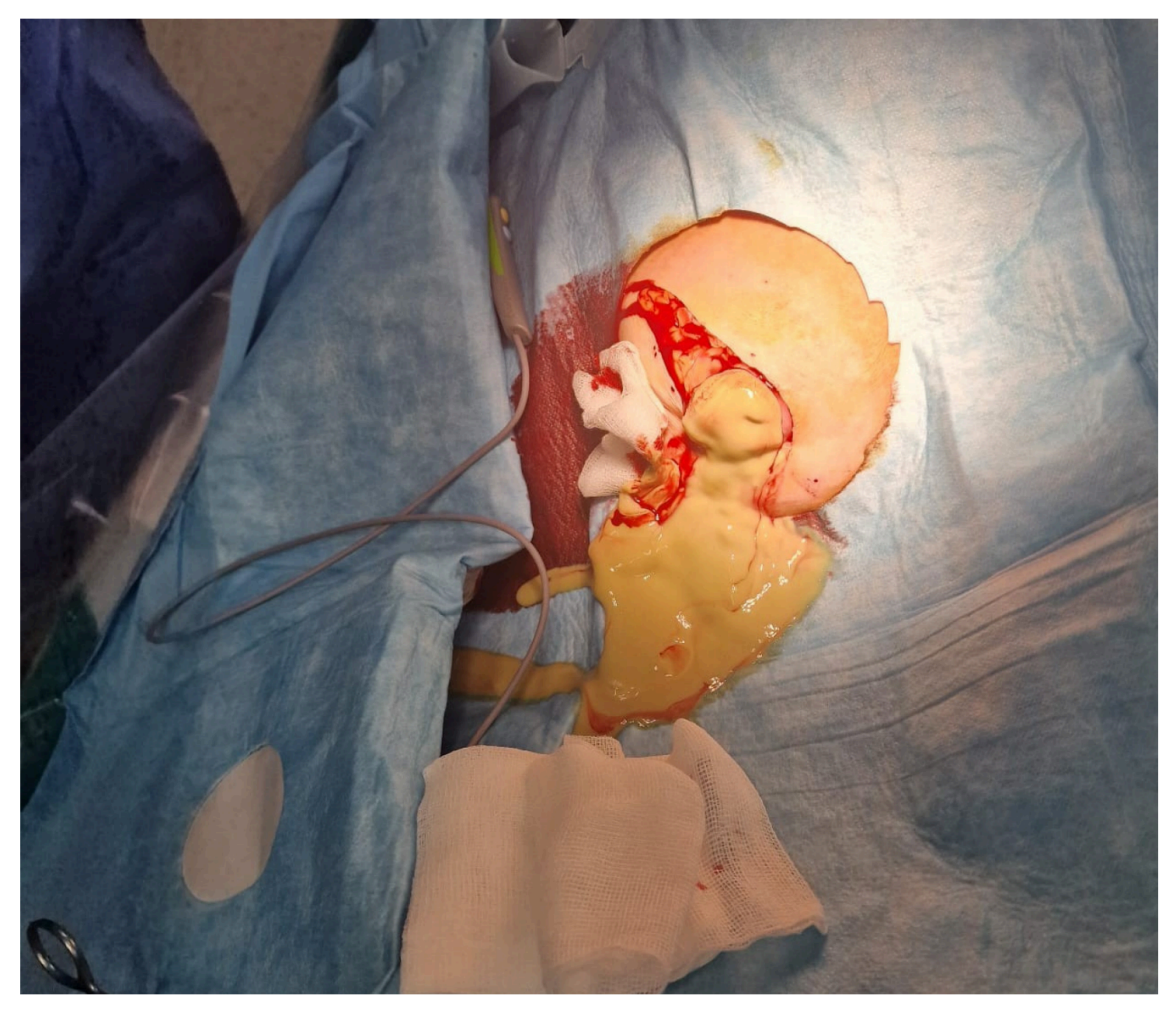
Intraoperative view. Upon skin incision, a large amount of odourless, thick, yellowish, creamy fluid drained spontaneously.

Given the progressive enlargement of the prepectoral mass in a patient with multiple risk factors, the surgical exploration of the pacemaker pocket was undertaken. Our objective was to accurately evaluate the local anatomy and to exclude a potential pocket colonisation, despite the lack of clinical and paraclinical evidence of infection. Upon incision of the pocket, the pacemaker and the proximal lead segments were found embedded in a large amount of odourless, thick, yellowish, creamy fluid that was evacuated (Figure 3).

**Figure 4:**
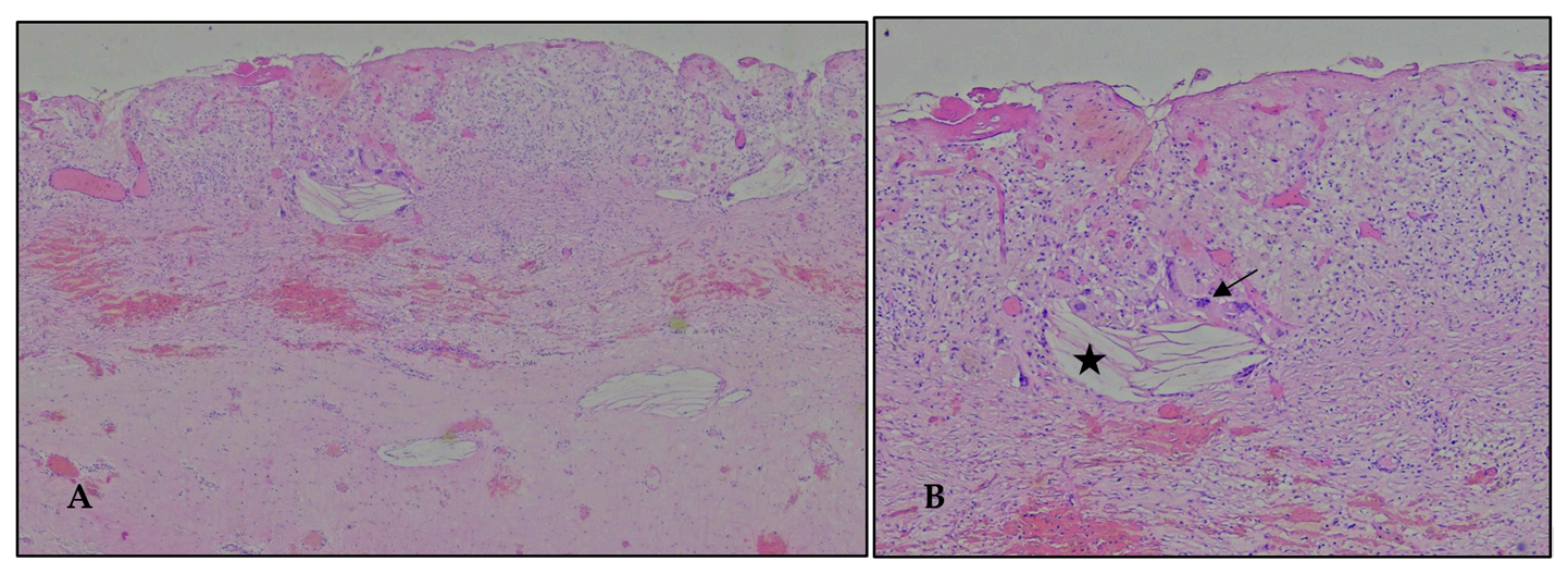
(**A**) Hematoxylin and eosin-stained histopathological image (5×) of the fibrous pseudocystic wall. (**B**) A 10× magnification detail, showing abundant inflammatory infiltrate: histiocytes, siderophages; multinucleated foreign-body giant cells (arrow); cholesterol crystals (star), suggestive of a granulomatous reaction.

The suture point of the leads was found to be inefficient, detached from the tissues, so the leads were no longer fixed. The whole mass, including the pacemaker and the leads, was surrounded by a mature fibrous capsule. A temporary transvenous pacing lead was introduced via the right internal jugular vein to ensure rhythm support during the diagnostic and decision-making interval. Both leads, although implanted four years earlier, were successfully removed by simple manual traction without the need for dedicated extraction tools. A partial capsulotomy of the pocket was performed, followed by thorough irrigation of the cavity. A surgical drain was placed, and layered wound closure was performed. Samples of the cyst wall and its contents were submitted for microbiological culture and histopathological assessment. Biochemical analysis could not be performed due to the thick, creamy consistency of the material. Also, microbiologic cultures from the fluid remained negative. Correlating the morphological appearance and immunophenotype of the fibrous cyst wall tissue, the conclusion was that of a chronic, nonspecific fibro-inflammatory granulomatous process (Figure 4).

**Figure 5:**
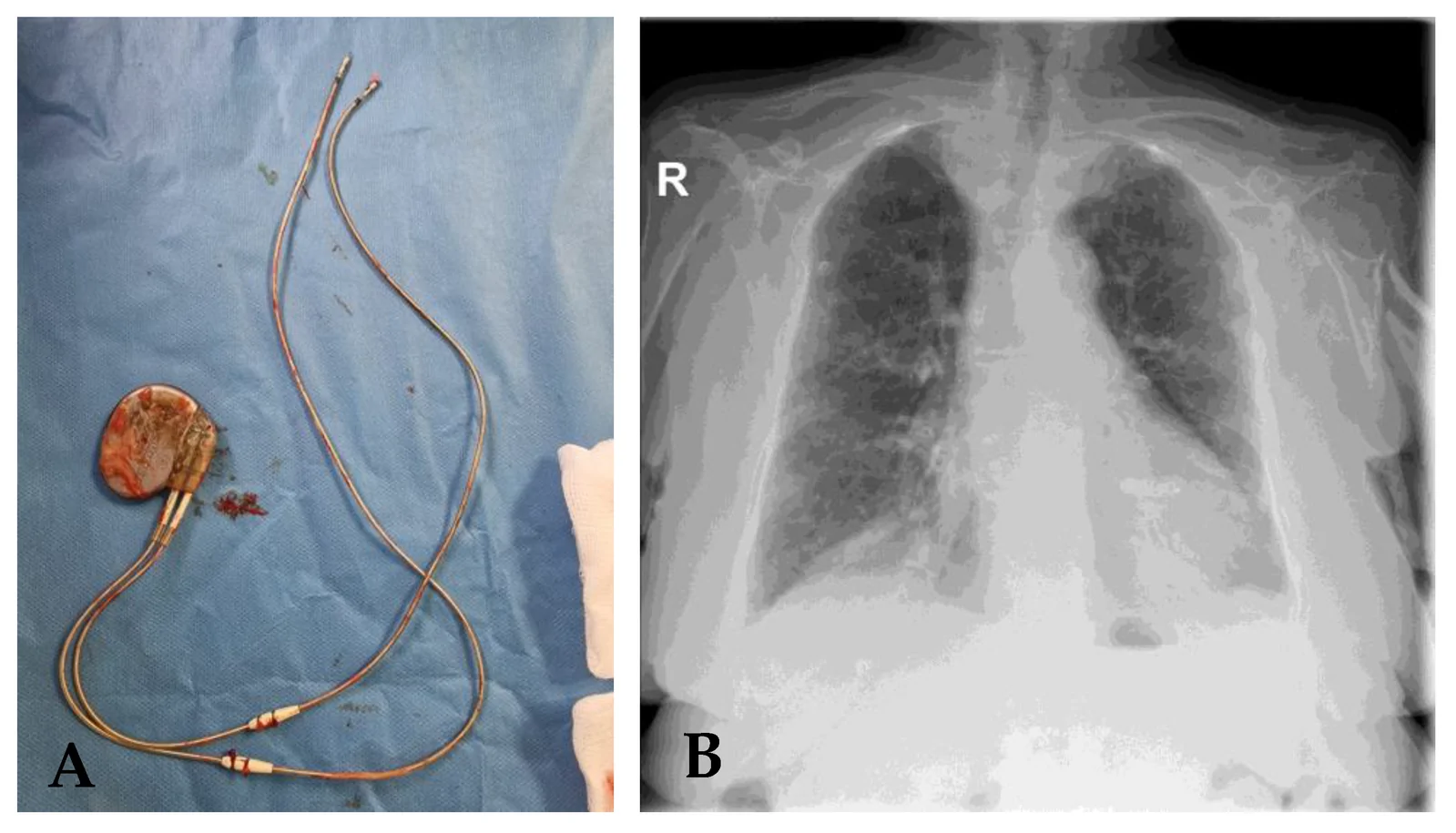
(**A**) The explanted pacemaker and four-year-old leads (by simple manual traction). (**B**) Posteroanterior (PA) chest X-ray showing the placement of a Micra™ VR, Medtronic, Minneapolis, MN, USA, leadless transcatheter pacemaker.

After the complete extraction of the entire transvenous pacing system (Figure 5A), implantation of a leadless transcatheter pacemaker (Micra^TM^ VR, Medtronic, Minneapolis, MN, USA) was performed, as it was considered the most appropriate long-term pacing strategy for this patient (Figure 5B).

The device was successfully implanted, with excellent acute pacing parameters and no procedural complications. A second transoesophageal echocardiography before hospital discharge did not detect intracardiac masses, nor pulmonary hypertension, and the tricuspid valve and right ventricular function were normal.

The patient progressed favourably, with no recurrence of swelling, no clinical signs of infection and stable device function at the 6-month follow-up.

## 3. Discussion

The patient presented a complex diagnostic challenge in the presence of bizarre enlarged swelling located at the pacemaker site with continuous and gradual growth.

### 3.1. Addressing a Swelling Pacemaker Pocket

**Figure 6:**
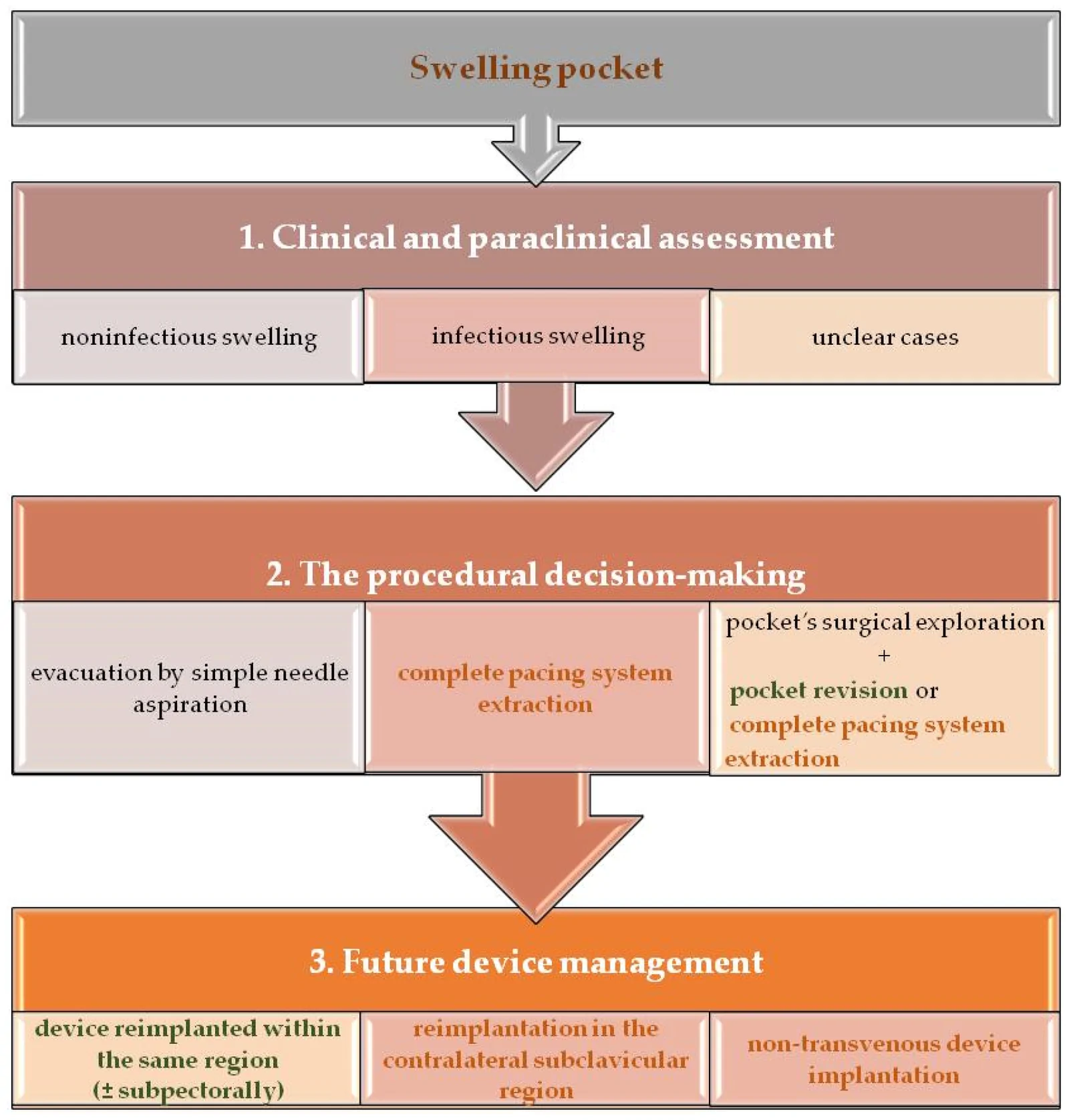
Flowchart illustrating the proposed strategic approach for managing a swelling pacemaker pocket in clinical practice.

Typically, in clinical practice, the strategic approach to a swelling pocket comprises three major stages (Figure 6).

#### 3.1.1. Clinical and Paraclinical Assessment

This non-invasive assessment is intended to guide the diagnosis towards the underlying cause (infectious or non-infectious).

In our case, the clinical picture lacked systemic infectious manifestations, inflammatory markers were normal, and the blood cultures were negative. Transoesophageal echocardiography revealed a filamentous structure on the atrial portion of the ventricular lead, most likely organised fibrin, but it had very little impact on therapeutic decision-making. It is well known that this imaging technique cannot reliably distinguish between infectious and non-infectious lead-related ultrasound densities [9,14,15]. There is growing evidence, in recent years, that not all lead-based echodensities are suggestive of CIED infections, and also their clinical significance remains insufficiently defined [16,17].

The imaging appearance of the voluminous encapsulated cystic lesion surrounding the pacemaker and proximal leads did not provide useful information to better define the etiology in this patient with seropositive rheumatoid arthritis, a history of infectious episodes, and repeated interventions in the pacemaker pocket area (lead and device extraction).

#### 3.1.2. The Procedural Decision-Making—Multiple Scenarios

At this stage, different clinical scenarios should be considered.

1. A doubtless diagnosis of infectious pacemaker pocket swelling constitutes a class I recommendation for transvenous lead extraction in both past and current consensus guidelines [9,13,14]. In this situation, pocket opening and drainage, complete pacing system extraction, and subsequent targeted antibiotic therapy tailored by an infectious disease specialist are required.

2. Although needle aspiration is generally discouraged to avoid the risk of infection and fluid reaccumulation, cases of non-infectious collections successfully managed with this approach have been reported in the literature [18,19]. Therefore, in those cases where an infectious etiology can be entirely ruled out, evacuation via simple needle aspiration—performed under strict aseptic conditions and followed by close clinical monitoring—may be sufficient and avoid the “capsulectomy” which may lead to significant pocket bleeding and determine the appearance of a possible haematoma [20].

3. In unclear cases with multiple risk factors, surgical exploration of the pacemaker pocket is the treatment of choice. Previous studies have shown that even in the absence of signs of a clinical infection, cultures taken at the time of CIED replacement have shown a significant incidence of pocket colonisation [21]. In addition, in the case of a potential infection, the fibrous capsule prevents the body’s defence mechanisms and antibiotic penetration [20]. This procedural stage is critical because adequate visualisation of the local anatomy is possible only after the pocket has been opened and drained. The appearance of the surrounding tissues is of paramount importance following the debridement of macerated or necrotic areas and the removal of blood clots. The local blood and lymphatic circulation must also be assessed. A variety of clinical factors can alter tissue structure and local circulation. Furthermore, these factors may predispose the site to postprocedural infection and include the following: age, diabetes mellitus, chronic kidney disease, rheumatologic diseases, and the number of prior surgical interventions in the same anatomical area [11,22,23,24]. At this stage, depending on local tissue findings and the distinct clinical characteristics of each case, an experienced operator is capable of immediately defining the subsequent therapeutic strategy.

#### 3.1.3. Future Device Management

This third stage is also crucial, as it entails the decision-making strategy for device reimplantation. This may be limited to pocket revision, with the leads maintained in situ and the device reimplanted within the same region, or potentially positioned subpectorally (this avoids tensioning the overlying skin, which is often compromised by the pathological process) [25,26]. Alternatively, in some cases, a safer approach may entail the complete extraction of the system followed by a traditional transvenous CIED implantation in the contralateral subclavicular region or by a non-transvenous device implantation.

In our case, surgical exploration of the pacemaker pocket was performed, and a significant amount of odourless, thick, yellowish, creamy fluid was drained off. Surgical exploration of the region revealed macerated tissues, with dislodgement of both the lead anchoring sleeves and the leads themselves. The regional anatomy was significantly altered, rendering the creation of a new pocket challenging and carrying a high risk of subsequent complications. Additionally, it was estimated that the leads could be easily extracted without posing a life-threatening risk to the patient. Due to the patient’s age, multiple comorbidities, and prior device-related infections, a conservative strategy avoiding subpectoral or contralateral pocket reconstruction was preferred, leading to the choice of a leadless pacemaker. Therefore, a complete extraction of the entire pacing system was performed through simple traction, without the use of specialised lead extraction tools, and in a second procedure, a leadless transcatheter pacemaker was implanted.

Leadless pacemakers (LPMs), subcutaneous (S-ICDs), and extravascular implantable cardioverter-defibrillators (EV-ICDs) are three outstanding alternatives to traditional CIEDs [27,28,29,30,31,32]. The rationale for the development of these new devices was focused on minimizing complications associated with traditional transvenous ones, to optimize patient outcomes, especially in high-risk patients. Their efficacy is already well established. Literature reports have consistently shown significantly lower rates of device-related infection with leadless systems compared with conventional transvenous systems, largely owing to the absence of a subcutaneous pocket and transvenous leads—components strongly associated with infection risk [27,28,33,34].

### 3.2. Non-Infectious CIED Pocket Swelling—A Parallel with the Current Literature

A fluid-filled, yellow, or creamy pacemaker pocket is a significant clinical finding that can sometimes indicate a serious infection, but it may also result from non-infectious causes.

Although much less common than early infectious etiologies, there are cases of possible non-infectious, delayed-onset CIED pocket swellings described in the literature including chronic expanding hematoma, seroma, lymphatic disruption or obstruction, foreign-body reaction, fat liquefaction, allergic reaction to the device components and less commonly, neoplastic processes [11,22,23,24]. Also, there are reports of late infective pocket swellings, which are considered secondary infections caused by blood-borne microorganisms [35,36]. Unlike patients with early infections (which are frequently women or under anticoagulant treatment and present local inflammation), those with late infections have more frequently CIED pocket skin erosion or valvular endocarditis [37].

The risk of CIED pocket complications is associated with a combination of predictors, including previous device infection, patient frailty, end-stage renal disease or renal insufficiency, diabetes mellitus, chronic obstructive pulmonary disease, corticosteroid use, malignancy, heart failure, pre-procedural fever and anticoagulant drug use [9]. Additional factors include the following: female gender; reduced body mass index (BMI); age over 80 years [2,3]; emergency admission; the complexity of device implantations such as ICD and/or cardiac resynchronization therapy procedures; system replacements or upgrades and lead revisions; low-volume operators (under 750 procedures/centre/year; under 50 procedures/operator/year) [38].

Non-infectious CIED pocket swellings have been described in only seven prior case reports (Table 1 and Table 2) [11,18,19,22,23,24,39].

There are many similarities to the case we are presenting: (1) the pocket swelling showed a slow and gradual progression; (2) in all of these cases, signs and symptoms of local or systemic infections were absent and inflammatory markers were negative; (3) most of the patients in whom these pocket lesions have been reported had multiple chronic conditions that increased the risk of complications; (4) all patients have negative blood cultures; (5) in six cases, the swelling was represented by cystic encapsulated lesions filled with viscous, odourless fluid [11,18,19,22,23,39]; (6) in four of these cases, pocket revision was performed [11,22,23,39] (in the other two cases, fluid collection was percutaneously drained [18,19]); (7) microbiological analysis (GRAM+, GRAM−, mycobacteria and anaerobic bacterial growth) ruled out the presence of pus.

Biochemical and/or cytological analysis of the fluid and the tissue biopsy from the fibrotic wall have highlighted different diagnoses. The seventh case was a very interesting, rare, squamous cell carcinoma of the lung spreading to the pacemaker pocket. Unfortunately, the patient refused further oncological investigations and died [24] (Table 2).

The available evidence is derived primarily from individual case reports and should consequently be interpreted with caution. Further reports and larger case series are needed to better characterize the clinical spectrum and establish optimal management strategies for this uncommon complication.

### 3.3. A Complex Diagnostic Challenge

In our case, histopathological analysis of the fibrous pseudocystic wall and its contents revealed abundant inflammatory infiltrate predominantly composed of histiocytes, siderophages, and multinucleated foreign-body giant cells, along with cholesterol crystals and rare lymphocytes and plasmocytes. Areas of microhemorrhage and multiple capillary vascular structures with marked stasis were also observed. All these suggest a chronic inflammatory reaction to necrotic tissue or old blood, with immune cells cleaning up debris, cholesterol crystals formed as a result of broken-down fat and tissue cells, and intense angiogenesis trying to heal the area, but with stasis, which often leads to further tissue congestion. The combination of this histological analysis with the patient’s sterile blood and fluid cultures, and the absence of systemic inflammatory response, suggested a non-infectious chronic process, the etiology of which remains uncertain in the absence of a biochemical analysis.

A precise distinction between an old liquefied hematoma, a chronic seroma, a chylous or pseudo-chylous fluid collection, and a foreign-body inflammatory tissue response cannot be definitively established. In fact, the histopathological characteristics of both the pseudocyst wall and the evacuated fluid in our patient are shared by all of these distinct etiologies. Therefore, the inability to perform a biochemical analysis to quantify triglycerides, cholesterol, and total protein constitutes a major diagnostic limitation in evaluating this patient.

Trends in the non-infectious complications following CIED implantation were investigated using a large population-based record-linkage study. The most common non-infectious swelling pocket complication was hematoma, which occurs in about 1.7% of cases, although incidences have trended down due to modern anticoagulation management [4,40,41]. At the beginning, in our case, an expanding hematoma was considered unlikely, given the absence of anticoagulant therapy and the long interval since pacemaker implantation. But, given the histopathological analysis’ result, an old liquefied hematoma can no longer be ruled out as the underlying cause of these pocket alterations.

Another potential cause of this pocket swelling could be a seroma, considering that seroma is defined as an abnormal accumulation of serous fluid composed of plasma, lymphatic fluid, and possibly inflammatory exudate within a dead space, with an etiology that remains incompletely understood [23]. It has been proposed that seroma formation results from disruption of vascular and lymphatic drainage following soft tissue dissection [42,43]. Seromas are associated with an increased risk of secondary infection and potential abscess formation [40]. Analyses of aspirated seroma fluid reported in the literature have demonstrated variable biochemical and cellular characteristics, with findings suggestive of both lymph-like fluid and inflammatory exudate [44]. In rare cases, chronic seromas may evolve into encapsulated pseudocysts requiring surgical drainage and debridement [45].

Furthermore, given the viscosity and macroscopic appearance of the drained material in a patient with rheumatoid arthritis, a collection of chylous or pseudochylous fluid secondary to disruption of the local lymphatic microcirculation could not be completely excluded, particularly in the context of repeated procedures in the same region. Chylous fluid collections have been described in two of the cases with atypical swelling of the CIED pocket, as presented above [19,39]. This diagnosis is very rare, with rheumatoid arthritis being listed among the possible causes [39]. However, in our case, the macroscopic appearance was not entirely consistent with that of chyle, which typically presents as a milky white fluid suggestive of high lipid or cholesterol content [18,19,46]. Once again, a biochemical analysis and the quantification of triglyceride levels would have been useful in more accurately defining the etiology.

Long-term implantation of medical devices into the body typically leads to a mild microscopic inflammatory and fibrotic process—the foreign body reaction—which rarely progresses to a massive, liquefied pseudocyst. In our patient, this exuberant, non-infectious chronic response was possibly driven by the synergy between her underlying rheumatoid arthritis and the tissue trauma from repeated local procedures [47,48].

### 3.4. The Particularity of the Case

This case highlights the complexity of decision-making in a large swelling pacemaker pocket when systemic signs of infection and systemic inflammatory response are lacking and the blood and fluid cultures are sterile, but the patient presents multiple risk factors for postprocedural infection and the etiology of non-infectious chronic process remains uncertain.

Non-infectious fluid collections are generally benign and managed conservatively, though persistent cases, especially when it causes severe symptoms, may require surgical revision and drainage. In addition, such late complications may closely mimic infectious processes, resulting in potential overtreatment including complete system extraction and represent a real diagnostic challenge. However, the presence of infection is not the only factor driving the decision to extract an entire system. Numerous other clinical conditions must be taken into account when determining the therapeutic strategy.

It is possible that in current practice, in many other cases, pocket swelling with this type of such a fluid aspect are mistakenly considered as having a common infectious etiology, not to be investigated further and not reported.

Through this case presentation, we aimed to propose a potential evaluation and decision-making algorithm for atypical scenarios. Furthermore, we sought to emphasise the importance of a precise local anatomical assessment and of the integration of all clinical, laboratory, and anatomical findings when arriving at this management decision.

The case also illustrates the value of leadless pacing systems as a viable and often preferable alternative in patients with prior device infection, significant inflammatory pocket reactions, or high infection risk profiles.

### 3.5. Limitations

Because the material drained from the pacemaker pocket was not liquid, but thick and creamy, biochemical analysis could not be performed in our laboratory to determine the level of triglycerides, cholesterol, and total protein and make a more specific differentiation between the various distinct etiologies that share the histopathological characteristics of both the pseudocyst wall and the evacuated fluid in our patient and this constitutes a major diagnostic limitation in evaluating this case.

The two leads, successfully removed by simple manual traction, did not present any tissue or thrombotic fragments that could be analysed to determine whether the filamentous structure on the atrial portion of the ventricular lead represented true organized fibrin. This assertion is supported by numerous documented cases of lead-associated ecodensities that occur in the absence of systemic infection.

Unfortunately, although we work in a large university hospital centre, there is no possibility to perform a lymphoscintigraphy, nor ^18^F-fluorodeoxyglucose positron-emission tomography/computed tomography (^18^F-FDG PET/CT), known as a validated multimodality whole-body technique that can identify infective foci, which may be useful in patients with CIED infection and help in decision making, especially in those with doubtful clinical presentation [49,50].

## 4. Conclusions

Non-infectious etiologies, although rare, should also be considered in the differential diagnosis of late-onset large fluid accumulation within the device pocket. In the absence of clinical or microbiological evidence of infection, conservative surgical management with pocket revision and capsulectomy may be sufficient, thereby avoiding unnecessary complete system extraction; however, the local tissue findings, after the pocket has been opened and drained, and the distinct clinical characteristics of each case, must ultimately define the therapeutic strategy. Careful integration of imaging, microbiology, and procedural findings is essential for accurate diagnosis. Balancing the patient’s pacing dependency, advanced age, history of infections, and an ambiguous inflammatory profile, combined with the successful extraction of the previous system, we concluded that implanting a leadless pacemaker offered the safest long-term solution in this case.

## Figures and Tables

**Table 1:** Clinical characteristics of patients with non-infectious CIED pocket swelling reported in the literature.

	Author Year	Country	Sex	Age (Years)	Implanted CIED Type	CIED Implantation Indication	Risk Factors for Pocket Complications
1	Bokhari [18] 2009	UK	F	80	PM DDD	sinus node disease	radiotherapy for left breast carcinoma
2	O’Neill [19] 2026	USA	F	58	PM DDD	third-degree AV block	myotonic dystrophy mild renal dysfunction hyperuricemia
3	Pai [11] 2022	India	M	70	PM DDD	2:1 AV block	diabetes
4	Schafer [23] 2024	Chile	M	72	PM DDD	ND	anticoagulation therapy
5	Guan [22] 2025	China	F	ND	PM DDD	ND	ND
6	Maffè [39] 2022	Italy	M	60	ICD DDD	DICMP (LVEF of 33%)	AP antibody syndrome rheumatoid arthritis
7	Popelier [24] 2022	Belgium	M	70	PM DDD	sick sinus syndrome	aggressive metastatic lung carcinoma

CIED, cardiac implantable electronic device; UK, United Kingdom; M, male; F, female; AV, atrioventricular; ND, Not Disclosed; PM, pacemaker; ICD, implantable cardioverter-defibrillators; DDD, dual-chamber pacing mode; DICMP, dilated ischemic cardiomyopathy; LVEF, left ventricular ejection fraction; AP, antiphospholipid; risk factors for pocket complications = multiple chronic conditions that increase the risk of complications at the CIED pocket site.

**Table 2:** Pocket lesion characteristics and outcomes of patients with non-infectious CIED pocket swelling reported in the literature.

	Author Year	Time	Pocket Revision	Fluid’s Aspect	Fluid Analysis	Pocket Lesion’s Etiology and Outcome
1	Bokhari [18] 2009	6 days	NO	white “milky” (60 mL)	chylous	Chylous fluid collection no further recurrence
2	O’Neill [19] 2026	6 years	NO	viscous, milky, odorless	chylous	Chylous fluid collection no further recurrence
3	Pai [11] 2022	5 years	YES	serosanguinous (100 mL)	NO	Seroma same PM was reinserted in the same region
4	Schafer [23] 2024	7 years	YES	yellowish-brown odourless (280 mL)	NO	Seroma (histopathological findings) favourable evolution
5	Guan [22] 2025	12 years	YES	thick yellow fluid	NO	Fat liquefaction (histopathological findings) favourable evolution
6	Maffè [39] 2022	7 months	YES	cloudy, gold-coloured	pseudochyle	Atypical hematoma containing chyle (histopathological findings) same ICD was reinserted in the same region
7	Popelier [24] 2022	9 months	NO	N/A (mass with increased vascularity)	N/A	Squamous cell carcinoma of the lung spreading to the pocket died after 1.5 months

Time = the time interval from CIED implantation to the development of pocket swelling; N/A, Not Applicable; PM, pacemaker; ICD, implantable cardioverter-defibrillators.

## Data Availability

The original contributions presented in this study are included in the article. Further inquiries can be directed to the corresponding author.

## Abbreviations

The following abbreviations are used in this manuscript:

BMI	body mass index
CIED(s)	cardiac implantable electronic device(s)
CRT-D	Cardiac Resynchronization Therapy-Defibrillator
CT	computed tomography
EV-ICDs	extravascular implantable cardioverter-defibrillators
^18^F-FDG PET/CT	^18^F-fluorodeoxyglucose positron-emission tomography/computed tomography
ICD	implantable cardioverter-defibrillator
LPM	Leadless pacemakers
MRSA	Methicillin-resistant Staphylococcus aureus
S-ICDs	subcutaneous and extravascular implantable cardioverter-defibrillators
